# Understanding the Radiobiology of Vestibular Schwannomas to Overcome Radiation Resistance

**DOI:** 10.3390/cancers13184575

**Published:** 2021-09-12

**Authors:** Torin P. Thielhelm, Stefania Goncalves, Scott M. Welford, Eric A. Mellon, Erin R. Cohen, Aida Nourbakhsh, Cristina Fernandez-Valle, Fred Telischi, Michael E. Ivan, Christine T. Dinh

**Affiliations:** 1 Department of Otolaryngology, University of Miami Miller School of Medicine, Miami, FL 33136, USA; torin.thielhelm@med.miami.edu (T.P.T.); stefania.goncalves@jhsmiami.org (S.G.); erco731@gmail.com (E.R.C.); axn644@med.miami.edu (A.N.); ftelischi@med.miami.edu (F.T.); 2 Department of Radiation Oncology, University of Miami Miller School of Medicine, Miami, FL 33136, USA; scott.welford@med.miami.edu (S.M.W.); eric.mellon@med.miami.edu (E.A.M.); 3 Burnett School of Biomedical Sciences, University of Central Florida College of Medicine, Orlando, FL 32816, USA; cfv@ucf.edu; 4 Department of Neurological Surgery, University of Miami Miller School of Medicine, Miami, FL 33136, USA; mivan@med.miami.edu

**Keywords:** vestibular schwannoma, radiobiology, ionizing radiation, radiation resistance, DNA damage, DNA repair, cell cycle, cell death

## Abstract

**Simple Summary:**

Vestibular schwannomas (VS) are intracranial tumors that originate from the Schwann cells of the vestibulocochlear nerve and cause hearing loss and dizziness. Although radiation therapy is a common treatment for VS, some irradiated tumors do not respond well and continue to grow, requiring additional therapies such as surgery. Little is known about the molecular mechanisms behind the normal response of VS to radiation therapy and why some VS are resistant to radiation. Thus, we aimed to review the current understanding of radiation response and resistance in VS through an in-depth summary of the DNA damage and cell cycle response to ionizing radiation. A better understanding of the radiobiology of VS can help guide future investigations looking at optimal radiation dosing strategies, unique targets for intervention, and novel therapies to improve patient outcomes.

**Abstract:**

Vestibular schwannomas (VS) are benign tumors arising from cranial nerve VIII that account for 8–10% of all intracranial tumors and are the most common tumors of the cerebellopontine angle. These tumors are typically managed with observation, radiation therapy, or microsurgical resection. Of the VS that are irradiated, there is a subset of tumors that are radioresistant and continue to grow; the mechanisms behind this phenomenon are not fully understood. In this review, the authors summarize how radiation causes cellular and DNA injury that can activate (1) checkpoints in the cell cycle to initiate cell cycle arrest and DNA repair and (2) key events that lead to cell death. In addition, we discuss the current knowledge of VS radiobiology and how it may contribute to clinical outcomes. A better understanding of VS radiobiology can help optimize existing treatment protocols and lead to new therapies to overcome radioresistance.

## 1. Introduction

Vestibular schwannomas (VS) are benign intracranial tumors that arise from the vestibulocochlear nerves. They can occur sporadically or as part of a genetic syndrome called Neurofibromatosis Type 2 (NF2). VS account for 8–10% of all intracranial tumors and are the most common tumor involving the cerebellopontine angle and internal auditory canal [1]. Although VS are benign, they can cause significant morbidity, including hearing loss, tinnitus, dizziness, and imbalance. Larger tumors can affect other cranial nerves, causing facial palsy, facial spasms, and facial numbness. As these neoplasms continue to grow, they can compress the cerebellum and brainstem, causing gait instability, narrowing of the fourth ventricle, hydrocephalus, transtentorial brain and tonsillar herniation, cortical infarctions, and death [2,3]. Intratumoral hemorrhage occurs in approximately 0.4% of VS, which can lead to rapid tumor growth and life-threatening complications [2,4]. Large VS can also cause papilledema and vision loss, diplopia, and vocal cord paralysis [2,5].

A majority of VS are sporadic, unilateral tumors (93%), while the remaining are associated with NF2 [6]. Sporadic VS are common, with a clinical prevalence that approximates 1 in every 2000 adults and 1 in every 500 individuals aged 70 years and older [6,7]. Treatment of sporadic VS can include initial observation with surveillance imaging, microsurgical resection, and/or radiotherapy [8]. Because NF2 can cause bilateral VS and other intracranial and spinal tumors, off-label chemotherapies have also been used for the treatment of VS in these individuals with partial success [9].

Initial observation with surveillance imaging is an appropriate treatment for many sporadic VS that have not grown large enough to cause symptomatic brainstem compression. The reasoning behind the “wait and scan” approach is to identify the patients that need active treatment for growing tumors in order to reduce treatment complications and improve quality of life for those that do not require active treatment [10]. In a meta-analysis of 2109 patients that received observation as the initial treatment, the local control rate was found to be 65% (95% Confidence Interval (CI): 55.9%, 73.6%) at the end of follow-up treatment (median: 3.4 years). Of those observed, 1560 patients had evaluations for serviceable hearing over time, and approximately 71.3% (95% CI: 52.9%, 86.6%) retained serviceable hearing at the end of the follow-up period [11].

Microsurgical resection of VS can be performed through three surgical approaches: middle cranial fossa, retrosigmoid, and translabyrinthine approaches [12]. In general, the middle cranial fossa approach is reserved for patients with smaller internal auditory canal tumors and serviceable hearing, as hearing preservation is possible through this corridor. The retrosigmoid approach is the most versatile technique, because it can be used for small to large tumors and allows broad visualization of the posterior cranial fossa contents and hearing preservation surgeries. The translabyrinthine approach entails opening the mastoid bone and drilling through the otic bone and thus results in complete hearing loss. The translabyrinthine approach can be used for small to large tumors; however, it is mostly used in patients with poor preoperative hearing. Although each surgical approach has its own advantages and disadvantages, there are risks that are unique to surgery and include inadvertent cranial nerve injury, persistent postoperative headache, cerebrospinal fluid leak, meningitis, brainstem stroke, and operative mortality [12,13].

Stereotactic radiosurgery (SRS) is a form of radiation where precise radiation doses can be delivered to the target tumor while minimizing radiation exposures to surrounding healthy structures, such as the cochlea and the brainstem [14,15]. There is significant heterogeneity in the radiation protocols used for VS, with modern protocols of radiation being delivered as a single fraction of ~11–13 Gray (Gy) or with biologically equivalent dosages in 3 to 5 fractions (“hypofractionated”) or in approximately 25 fractions (“fractionated”) [16,17,18]. Because of the broad range of radiation algorithms; differences in tumor size, tumor volume, and length of follow-up between studies; and the paucity of prospective investigations, it is particularly difficult to measure long-term treatment outcomes as it relates to observation and microsurgery.

Overall, tumor control after single fraction SRS is excellent, with long-term tumor control rates of around 88–91% [19,20]. Potential side effects of SRS include trigeminal neuropathy, permanent facial weakness, vertigo, and gait imbalance [21]. In addition, the hearing preservation rates decrease over time, with approximately 25% of VS patients maintaining serviceable hearing by 10-year follow-up [19,22]. Approximately 23–44% of irradiated VS have a transient increase in tumor volume 6 to 18 months after SRS, an event termed “pseudoprogression” [23,24,25]. Because pseudoprogression can worsen brainstem compression and cause hydrocephalus and ataxia, SRS is often limited for small-to-moderate sized tumors without significant mass effect on the brainstem [16,23,25]. A rare, potentially life-threatening, and delayed complication is radiation necrosis of the brain [26]. Radiation may also cause malignant degeneration of VS or secondary malignancies in the radiation field, a problem that is particularly concerning for younger patients [27,28,29].

Approximately 9–12% of VS patients that receive radiation develop tumor progression over time [19,20]. However, its arguable that the failure rates appear low because most VS were probably not growing at the time of radiation. The tumor control rate after single fraction SRS in growing VS has been reported to be approximately 77% at 10 years [30]. There is also increasing evidence that SRS is less successful for VS that exhibited faster growth rates (e.g., >2.5 mm/year in diameter or volume doubling time >15 months) [30,31,32]. In addition, the control rate approaches 80% for tumor volumes >6 cm^3^ [33], with tumor volume >15 cm^3^ as a significant predictor of radiation failure [23]. With single fraction SRS, published long-term tumor control rates for NF2-associated VS are about 84–87% [34,35,36]; however, the tumor control rate declines to about 40% for NF2 patients in some studies depending on the initial tumor size treated, the radiation protocol used, and whether the tumor was growing at the time of radiation [32,37].

It is particularly important to clinicians and surgeons to understand these nuances when determining which VS patients would benefit from initial radiation treatment, as salvage surgery after failed irradiation has increased morbidity. Radiation can make surgical resection more challenging by creating adhesions and fibrosis between the tumor and adjacent neurovascular structures, such as the facial nerve, brainstem, and cerebellar arteries [38]. Thus, the risk of postoperative complications is higher with irradiated VS, making the feasibility and rate of complete tumor resection lower [39,40].

The main goal of ionizing radiation (IR) in treating VS is to halt tumor growth and reduce tumor burden, while minimizing radiation toxicity to healthy surrounding neurovascular structures. Another important goal is to select VS patients that have the highest chance of tumor control or regression with radiation. To be able to achieve these goals, a thorough understanding of the radiobiology and mechanisms of radiation resistance in VS cells is needed. However, little is known about the biological effects of IR on VS cells, why some cells resist radiation more than others, and how radiation dose and fraction affect outcomes.

We review the general mechanisms of radiation-induced cellular and DNA injury and their impact on the cell cycle, cell death, and DNA repair pathways. We also summarize current knowledge regarding the effects of radiation on VS cells and describe future directions of research that can potentially improve clinical outcomes after radiation in VS patients.

## 2. DNA Damage after Ionizing Radiation

### 2.1. DNA Oxidation and Oxidative Stress

IR can cause DNA damage through direct and indirect mechanisms [41,42,43,44]. Specifically, IR can injure nucleotides directly through one-electron oxidation of DNA, typically causing guanine damages that can be detected as 8-oxo-7,8-dihydro-2′-deoxyguanosine (8-oxodGuo) [43,44]. IR can damage DNA indirectly through ionization of cellular water and generation of reactive oxygen species (ROS), such as hydroxyl radicals (^−^OH), as well as formation of secondary ROS and reactive nitrogen species (RNS). The oxidative stress caused by ROS and RNS can cause base damages, DNA breaks, complex DNA damage lesions, as well as injury to cellular proteins and lipids [44]. In response to IR-induced alterations in DNA, specific sensor proteins detect DNA damage, initiate downstream signaling that activate cell cycle check points, induce cell cycle arrest, and recruit additional proteins to repair DNA [45]. In the following section, we focus on early cellular responses to IR-induced single-stranded breaks (SSBs), double-stranded breaks (DSBs), and more complex DNA damage lesions and ways to detect DNA damage.

### 2.2. DNA Single-Strand Breaks

SSBs are the most common type of DNA damage and are characterized by discontinuity of one strand of the DNA double helix. SSBs occur when a single nucleotide is lost and there is injury at the 3’ or 5’ end of the break [46,47,48]. Overall, SSBs are seldom lethal, as cells have evolved mechanisms to repair these DNA lesions [46,49]. Base excision repair (BER) is the most common method of repairing oxidized bases and SSBs [50]. However, in certain situations, SSBs can result in cell death through various mechanisms [51]. In nonproliferating cells, an acute increase in SSBs can saturate the DNA repair system and activate poly (ADP-ribose) polymerase 1 (PARP1), the SSB sensor. Prolonged activation of PARP1 leads to the depletion of nicotinamide adenine dinucleotide (NAD) and adenosine triphosphate (ATP), which stimulates the mitochondrial release of apoptosis-inducing factor (AIF) into the cytoplasm to initiate caspase-independent apoptosis [52]. In proliferating cells, SSBs lead to collapse of the DNA replication fork at the break during the S phase of the cell cycle; this event can trigger cell cycle arrest. In addition, when SSBs are not repaired, mutations accumulate leading to further lethal DNA damage and carcinogenic transformation [46,52].

### 2.3. DNA Double-Strand Breaks

DSBs in DNA are less common after IR than SSBs but can be lethal to cells by introducing genetic instability and promoting cell death. Therefore, cellular response machinery must act quickly to recognize DSBs and activate the DNA damage response (DDR) system to effectively repair this insult [53]. The MRN protein complex (Meiotic Recombination 11 (MRE11), RAD50, and Nibrin (NBN or NBS1)) plays a crucial role in the DDR by recognizing DSBs, inducing cell cycle arrest, repairing damaged DNA, and resuming the cell cycle once DNA repair is completed [54].

When DSBs are present in the G1 phase of the cell cycle, MRE11 and NBS1 detect the DSB, MRE11 induces autophosphorylation and monomerization of the ataxia telangiectasia mutated (ATM) kinase, and ATM kinase stabilizes tumor protein 53 (p53), which leads to cell cycle arrest [55]. When DSBs occur in the S or G2 phase, NBS1 activates ATR Rad3-related (ATR) kinase, which signals downstream events that lead to cell cycle arrest [56].

Subsequently, RAD50 forms a complex with MRE11 that functions as a scaffold to align break ends and facilitate repair. ATM and ATR phosphorylates Ser-139 of the H2A histone family member X (H2AX), converting it to gamma-H2AX [53,54,57]. Gamma-H2AX activates the DDR system and, with the help of MRE11, initiates DNA repair [53,58] Furthermore, ROS produced by IR can directly induce autophosphorylation of ATM, activating it and initiating the DDR system [53,59,60,61].

### 2.4. Oxidative Clustered DNA Lesions

Clustered DNA lesions are a recognized form of IR-induced DNA damage. These lesions are a combination of multiple lesions resulting from a single radiation track passage. Clusters can include SSBs, DSBs, oxidized or abasic sites, and complex DNA crosslinks [62]. In fact, simple DSBs can be considered a type of clustered DNA lesion, as DSBs are simply two SSBs on opposing strands. Overall, these lesions are referred to as oxidative clustered DNA lesions (OCDLs), which are closely spaced DNA lesions of up to 10 base pairs [63]. OCDLs are difficult for the cell to repair, and ongoing research is targeted at elucidating the complex DNA repair mechanisms associated with these more recently discovered DNA damage lesions.

### 2.5. Markers of DNA Damage

DNA damage detection in the laboratory can be performed using analytical chemistry to directly identify oxidative changes such as pyrimidine base dimers in the DNA structure. Unfortunately, these are usually not specific to the type of insult, making it difficult to assess the damaging effect of a particular agent. Therefore, scientists have focused on designing laboratory-engineered antibodies that detect specific DNA structural changes or the presence of repair enzymes [41].

Newer and more common DNA damage biomarkers are being used to determine activation of the DDR system. The most commonly targeted DDR proteins are: p53, gamma-H2AX, checkpoint kinase 1 (Chk1), ATM and ATR protein, p53 binding protein 1 (TP53BP1), caspase-3 (CASP3), MRE11, and catalytic subunit of the DNA-dependent protein kinase (DNA-PK) [41]. In particular, gamma-H2AX is a very sensitive and reliable marker and is considered the gold standard for detecting radiation-induced DSBs. In immunofluorescence, one nuclear focus of gamma-H2AX correlates to one DSB. Other proteins can be utilized to measure the levels of DSBs; however, these other proteins can be more difficult to measure using focus analysis [41,64,65]. Other biomarkers that can be used to study the effects of IR are damage-specific DNA binding protein 2 (DDB2), NBS1, Chk2, X-ray repair cross complementing 1 (XRCC1), and RAD51 [41].

Because DSBs are lethal lesions that can lead to chromosomal instability with unfavorable effects on cell survival, we focus this review primarily on the downstream events after radiation-induced DSBs and the activation of repair mechanisms.

## 3. DNA Repair after Ionizing Radiation

Normal cells have different mechanisms for immediate recognition and repair of DNA damage to restore and preserve chromatin architecture and normal cell homeostasis [53]. Different DNA repair pathways have been identified, such as: (1) BER, which targets damaged bases and SSBs; (2) mismatch repair, which corrects mispaired nucleotides; (3) nucleotide-excision repair, which removes a variety of helix-distorting DNA lesions; (4) homologous recombination (HR) and nonhomologous end joining (NHEJ), which repair DSBs; and (5) translesional synthesis, which bypasses DNA adducts (segments of DNA bound to a cancer-causing chemical) during DNA replication [49,66]. We focus primarily on HR and NHEJ, since they are the primary mechanisms of DNA repair following the development of DSBs after IR [67,68].

### 3.1. Homologous Recombination

**Figure 1:**
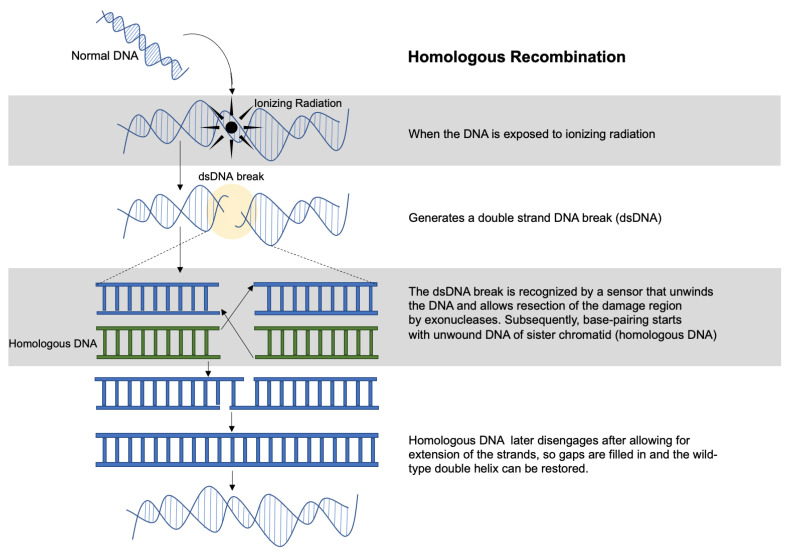
Diagram of homologous recombination after ionizing radiation. Ionizing radiation can initiate double-stranded DNA (dsDNA) breaks that can be repaired through homologous recombination—a process in which the cell utilizes a homologous DNA strand as a template for DNA repair.

HR is an essential method of repairing DNA DSBs through the alignment of homologous sequences of DNA (Figure 1), mainly during the S to G2 phases of the cell cycle [69]. HR also allows for recovery from DNA damage by assisting with DNA replication when replication forks are not properly functioning [70]. In HR, RAD51, a RecA homolog, is the main protein involved in identifying homologous strands and opening and unwinding the DNA to allow DNA repair proteins to enter [71]. However, other proteins such as RAD50, MRE11, NBS1 and breast cancer type 1 susceptibility protein (BRCA1) can be involved [49,66]. In HR, DNA exonucleases and helicases resect the DSB in a 5′–3′ direction during the S or G2 phases to create single-stranded DNA (ssDNA) 3′ overhangs. The 3′ overhang subsequently activates ATR, which initiates a complex process that repairs the DSB using complementary DNA as a template [54,72]. The use of a complementary strand makes this pathway a highly efficient and error-free DNA repair system [53].

### 3.2. Nonhomologous End-Joining Recombination

**Figure 2:**
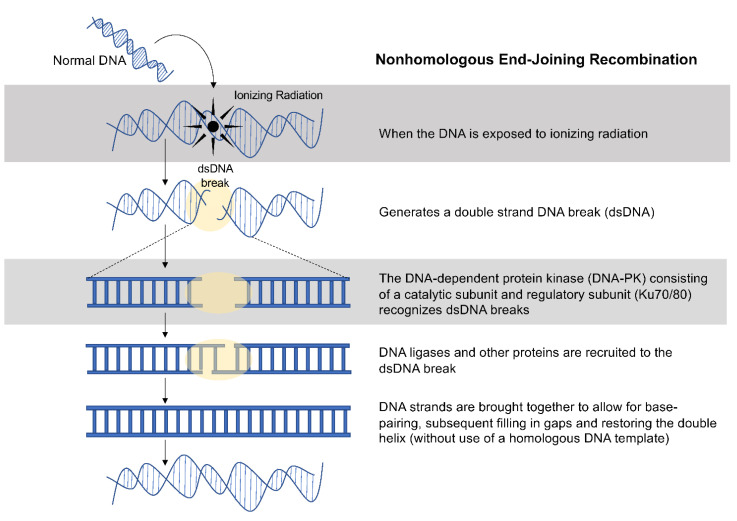
Schematic representation of nonhomologous recombination after ionizing radiation. Nonhomologous recombination is a method of DNA repair that can occur following ionizing radiation. In this process, DNA-dependent protein kinase (DNA-PK) recognizes double-stranded DNA (dsDNA) breaks and recruits other proteins to repair the DNA injury without the use of a homologous DNA template.

The classical NHEJ pathway is highly conserved and employed within cells throughout the cell cycle. Contrary to HR, NHEJ is prone to error because it does not use a complementary DNA template to repair DSBs. Without the use of a complementary DNA template, base pairs can be inserted or deleted [41,53,71]. In the classical pathway, the catalytic subunit of DNA-PK, bound to the Ku70/80 heterodimer, is responsible for detecting DSBs, attaching to the ends of DNA strands, and recruiting enzymes that ligate broken ends together (Figure 2) [73,74]. This process involves several protein complexes, including DNA ligase IV, XRCC4, and XLF/Cerrunnus [75]. In addition, RAD50 and MRE11 form a complex that can hold free DNA ends together during the repair process. Because classical NHEJ does not require DNA sequence homology, DNA repair often results in the joining of unrelated DNA segments [73,76,77].

The alternative NHEJ pathway allows for DNA repair without the classical NHEJ proteins. This process involves the ligation of exposed microhomologous sequences and is known as microhomology-mediated end joining (MMEJ). Important proteins associated with MMEJ include PARP1, ATM, MRE11, and C-terminal-binding protein 1-interacting protein (CtIP) [73,78].

## 4. Cell Death after Ionizing Radiation

Following exposure to IR, the accumulation of ROS and free radicals leads to DNA damage; if the damage is not repaired, the cell undergoes cell death [79]. There are several mechanisms of IR-induced cell death, which include apoptosis, necrosis, autophagic cell death, and mitotic catastrophe (MC). Cell type, radiation dose, and fractionation protocols may determine the specific cell death pathway that is activated.

Specifically, IR can induce apoptosis through p53-induced transcription of proapoptotic proteins in the mitochondrial-dependent intrinsic pathway, but it can also promote apoptosis through the upregulation of death receptors in the extrinsic pathway [80,81]. In addition, IR-induced activation of ATM may play a role in initiation of necroptotic pathways [82,83]. Persistent DNA damage from IR can also induce p53 and ATM to activate autophagic cell death [80,81,83,84]. Furthermore, irradiated cells can undergo MC before initiating apoptosis or necrosis by prematurely entering mitosis before repair of DNA damage [85]. IR can also initiate a state of prolonged growth arrest termed tumor senescence, which is an alternate cell fate where cells permanently lose proliferative capacity [86].

**Figure 3:**
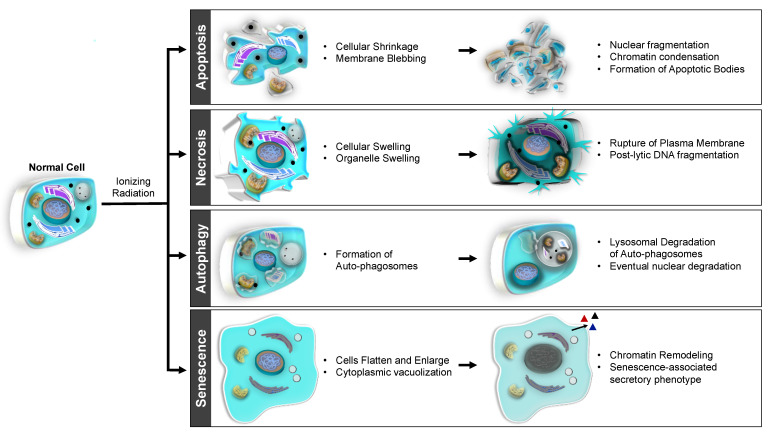
Radiation-induced cell death through apoptosis, necrosis, autophagy, and senescence. Apoptosis is a form of programmed cell death that involves cell body shrinkage, membrane blebbing, nuclear fragmentation, chromatin condensation, chromosomal DNA cleavage, and phagocytosis of the resulting apoptotic bodies. Necrosis is a less regulated form of cell death that involves organelle and cellular swelling, plasma membrane rupture, and leakage of cellular contents with DNA fragmentation. Autophagic cell death involves the lysosomal degradation of double-membraned vesicles called autophagosomes, which contain organelles and cellular contents. Senescence is a state of prolonged cell cycle arrest with permanent loss of proliferative potential, characterized by flattening and enlargement of cells, cytoplasmic vacuolization, chromatin remodeling, and secretion of senescence-associated secretory phenotype. Adapted from Dinh, C.T.; Goncalves, S.; Bas, E.; Van De Water, T.R.; Zine, A. Molecular regulation of auditory hair cell death and approaches to protect sensory receptor cells and/or stimulate repair following acoustic trauma. *Front. Cell. Neurosci.* **2015**, *9*, 96 [87].

A review of general concepts in apoptosis, necrosis, autophagic cell death, MC, and cellular senescence is offered below. Morphologic changes in cells typical of apoptosis, necrosis, autophagic cell death, and senescence are illustrated in Figure 3.

### 4.1. Apoptosis

Apoptosis, a main death modality known to occur after IR, is a form of programmed cell death involving cell body shrinkage, membrane blebbing, nuclear fragmentation, chromatin condensation, and chromosomal DNA cleavage. The resulting apoptotic bodies are digested via phagocytosis. Apoptosis can occur through the intrinsic or extrinsic signaling pathways. The intrinsic pathway, which accounts for the majority of radiation-induced apoptosis, is mitochondria dependent, while the extrinsic pathway is mediated through activation of death receptors [51,83,87].

#### 4.1.1. Intrinsic Pathway

In the intrinsic pathway, DNA damage activates p53 to promote transcription and translation of the proapoptotic proteins Bcl2-associated X (BAX) and Bcl-2 antagonist/killer 1 (BAK1) [51,88]. BAX and BAK1 proteins form pores at outer and inner mitochondrial membranes and promote mitochondrial outer membrane permeabilization (MOMP). Subsequently, proapoptotic proteins are released from the mitochondrial intermembrane space into the cytoplasm [83]. Important apoptotic factors that are released in this process include cytochrome c (cyt c), apoptosis inducing factor (AIF), second mitochondria-derived activator of caspases/direct inhibitor of apoptosis protein binding protein with low pI (SMAC/DIABLO), mammalian homolog of bacterial high temperature requirement protein A2 (Omi/HtrA2), and endonuclease G (EndoG). These proteins can initiate downstream events that lead to apoptosis in a caspase-dependent or caspase-independent manner [89].

#### 4.1.2. Extrinsic Pathway

In contrast to the intrinsic pathway, extrinsic apoptosis is accomplished through activation of plasma membrane death and dependence receptors by ligands that are stimulated by an extracellular signal [83]. IR can promote extrinsic apoptosis by injuring DNA and promoting p53 activity, which leads to downstream activation of death receptors and associated pathways [90]. Commonly studied death receptors include FAS (CD95), TNFR1, TRAMP (DR3), TRAILR1 (DR4), TRAILR2 (DR5), and DR6. When death ligands (e.g., FasL, TNFa) bind their associated death receptors, a death-inducing signaling complex (DISC) assembles, allowing for recruitment of adapter molecules (including Fas-associated death domain protein (FADD)) and activation of caspase-8 and -10 [91]. Caspase-8 is responsible for subsequent activation of the executioner caspases, namely caspase-3 and -7, which initiate programmed cell death mechanisms as a result of extracellular signals [92]. More recently, the extrinsic pathway has been shown to be activated through dependence receptors when associated ligand levels drop. Examples of dependence receptors include DCC, Neogenin, and RET [93].

### 4.2. Caspase-Dependent Cell Death

In caspase-dependent intrinsic apoptosis, cyt c forms a supramolecular complex called an apoptosome with apoptotic protease-activating factor-1 (Apaf-1) and procaspase 9 [94]. The apoptosome activates caspase-9, which can then catalyze the activation of caspase-3 and -7 [95]. In extrinsic apoptosis, activation of death receptors promotes caspase-8 activity, which will then catalyze the activation of caspase-3 and -7 [92]. Once caspase-3 and -7 are activated, caspase-activated DNase (CAD) fragments the DNA, apoptotic chromatin condensation inducer in the nucleus (ACINUS) initiates chromatin condensation, and cleaved HELI-CARD (helicase with an N-terminal caspase-recruitment domain) accelerates DNA degradation [96,97,98]. Smac/DIABLO and Omi/HtrA2 are proteins that can initiate caspase-dependent apoptosis by binding to X-linked inhibitor of apoptosis protein (XIAP) and releasing XIAP-inhibition of caspase-3, -7, and -9 [99].

### 4.3. Caspase-Independent Cell Death

In the intrinsic pathway, caspase-independent apoptosis can be induced when EndoG migrates to the nucleus and cleaves chromosomal DNA. Similar to EndoG, AIF, a mitochondrial protein, migrates from the cytosol to the nucleus. Once in the nucleus, AIF promotes chromatin condensation and DNA fragmentation in a distinct mechanism from apoptosis termed parthanatos, which is a PARP-1-dependent cell death pathway [100,101]. Additionally, Omi/HtrA2 can result in caspase-independent cell death via its serine protease activity, but the exact details of this pathway are unknown [102,103].

### 4.4. Necrosis

While low doses of IR have been associated with apoptosis, higher doses of IR can lead to necrosis [104]. The exact mechanisms of how IR induces necrosis are not fully understood, but studies suggest that IR can activate ATM, which results in necrosis in the absence of caspase-8 activity. In contrast to apoptosis, necrosis involves organelle swelling, increased cell volume, plasma membrane rupture, and subsequent leakage of cellular contents into the extracellular space with DNA fragmentation. The downstream events of necrosis involve the accumulation of mitochondrial ROS which leads to the induction of mitochondrial permeability transition (MPT) via the opening of the permeability transition pore complex (PTPC) [83,105,106]. During MPT, the inner mitochondrial membrane becomes more permeable, which results in water transfer into the mitochondrial matrix. The outer mitochondrial membrane then swells and ruptures. This process requires cyclophilin D (CypD), an integral part of the PTPC for MPT-dependent necrosis [107,108].

Necroptosis is a form of necrosis but represents a more regulated form of necrotic cell death [83,109]. In necroptosis, IR causes DNA damage and ATM activation, which initiates the action of receptor interacting protein kinases (RIPK). Specifically, RIPK3 complexes with RIPK1 in a necrosome, which then initiates the cascade of necroptosis [110,111]. The process is also regulated by the expression of pseudokinase MLKL (mixed lineage kinase domain-like protein), which is a critical substrate of RIPK3. Phosphorylation of RIPK3 initiates the phosphorylation of MLKL, which leads to plasma membrane rupture during necroptosis by mediating sodium influx through Ca^2+^ and Na^+^ ion channels [112,113,114].

### 4.5. Autophagic Cell Death

Persistent DNA damage from IR can induce activation of ATM, which triggers the cellular self-degradation known as autophagic cell death [115]. Normally, mammalian target of rapamycin complex 1 (mTORC1) activity prevents autophagic cell death by blocking Unc-51 Like autophagy-activating kinase 1 (ULK1) activation [116]. After IR, ATM activates AMP-activated protein kinase (AMPK) which inhibits mTORC1. This leads to the formation of autophagosomes, which are double membraned vesicles formed by autophagy-related (ATG) proteins. The formation of autophagosomes requires ULK1, ATG13, FAK-interacting protein FIP200, ATG101, and over 15 other ATG proteins. In addition, inhibition of mTORC1 releases the ULK1 blockade, which is a critical step for autophagosome activation and downstream lysosomal degradation of proteins and organelles [116,117,118].

### 4.6. Mitotic Catastrophe

MC is a mechanism of cell death occurring during or after aberrant mitosis. MC can occur where cells with unrepaired DNA damage enter mitosis prematurely [85]. Irradiated cells that are unable to activate cell cycle checkpoints, enter cell cycle arrest, and/or repair DNA may undergo MC. The end result of MC is the generation of nuclear envelopes that surround aberrantly segregated chromosomes and the initiation of premature chromatic condensation and DNA fragmentation [85]. Once MC occurs, cells can undergo cell death via apoptosis or necrosis pathways. The mechanisms behind MC are not fully understood and there is some evidence to suggest that MC is a process that precedes apoptosis and necrosis [85,119,120,121].

### 4.7. Cellular Senescence

IR can induce cellular senescence, which is a state of prolonged growth arrest with permanent loss of proliferative potential [122]. Although the exact mechanisms are unknown, cellular senescence is believed to occur after IR-induced DSBs are detected, the DDR system is activated, and p53 and/or other cyclin-dependent kinase (CDK) inhibitors, such as p21 and p16, accumulate [123]. Expression of p16 inhibits CDK4 and CDK6, hypophosphorylates retinoblastoma protein (Rb), and blocks cells from entering S phase to mediate permanent cellular rest. The p16–Rb pathway can increase cellular ROS, activating protein kinase Cdelta (PKCdelta) to generate more ROS in a positive feedback loop to sustain PKCdelta activity. It is thought that sustained activation of PKCdelta blocks cell proliferation irreversibly [124]. Senescent cells may flatten and appear enlarged, develop cytoplasmic vacuolization, and undergo large-scale chromatin remodeling [125]. Senescent cells also produce and secrete a complex mixture of cytokines, chemokines, proteases, growth factors, and other signaling molecules, termed senescence-associated secretory phenotype (SASP) [126]. SASP initiates an autocrine positive feedback loop that supports senescence growth arrest. Senescent cells also undergo metabolic changes, such as mitochondrial metabolism to maintain SASP production [127]. The most common senescence marker is senescence-associated β-galactosidase (SA β-gal) [128].

## 5. Cell Cycle after Ionizing Radiation

Irradiated cells can evade DSB-induced programmed cell death by activation of cell cycle checkpoints, entering cell cycle arrest, and repairing DNA damage [79]. To understand how radiation can initiate cell cycle arrest to repair DNA, we review how cells progress through the normal cell cycle and activate cell cycle checkpoints in response to radiation.

### 5.1. Normal Cell Cycle

**Figure 4:**
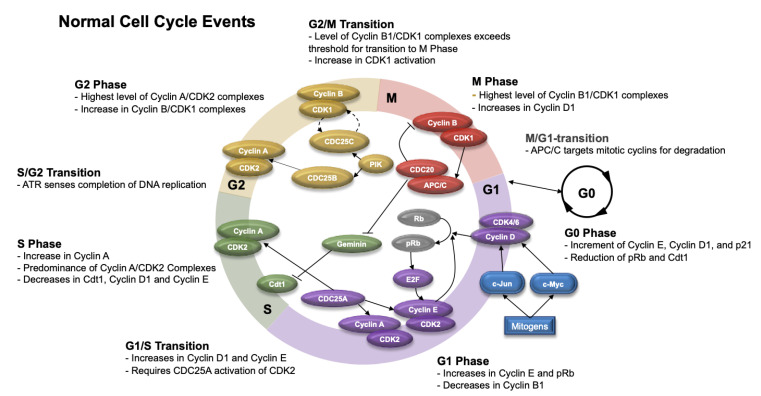
Diagram of the normal cell cycle. This diagram shows the G0, G1, S, G2, and M phases of the cell cycle, which are characterized by expression of various cyclins and proteins. Checkpoints exist throughout the cell cycle to ensure DNA integrity. APC/C (anaphase promoting complex), ATR (ataxia-telangiectasia and Rad3-related protein), CDC25 (cell division cycle 25), CDK1/2/4/6 (cyclin-dependent kinase 1/2/4/6), Cdt1 (chromatin licensing and DNA replicating factor 1), E2F (family of transcription factors), PIK (phosphatidylinositol kinase), pRB (phosphorylated retinoblastoma protein), Rb (retinoblastoma protein).

The cell cycle consists of several phases, which include G1 (gap 1), S (synthesis), G2 (gap 2), M (mitotic), and G0 (Gap 0). In the G1 phase, the cell grows, and cellular contents actively replicate. When the cellular environment is appropriate for DNA replication, the cell enters into the S phase. In S phase, DNA synthesis occurs, and the genetic content duplicates. Subsequently, the cell enters into the G2 phase, when protein synthesis and cell growth occur in preparation for mitosis. In the M phase, the cell divides and distributes its DNA and cytoplasm to produce two individual cells. Afterwards, the cell returns to the G1 phase or, in certain circumstances, can enter into the G0 phase. The G0 phase is a resting phase where the cell exits the cell cycle and either divides or prepares to divide [129,130]. Figure 4 highlights the phases of the cell cycle and its cell cycle checkpoints.

The retinoblastoma protein (Rb) is an important regulator of the cell cycle. In the G1 phase, uncommitted cells, Rb (in its unphosphorylated form) binds to the E2F transcription factor and forms an inhibitor complex with histone deacetylase to repress downstream transcription activities [131]. When this occurs, the cyclin-dependent kinases (CDKs) are inactive. Upon receiving extracellular mitogenic signals, transcription factors such as c-Myc and c-Jun become activated, upregulating Cyclin D. Subsequently, Cyclin D binds CDK4 and CDK6, which phosphorylates Rb (to pRB) and results in unbinding and activation of the E2F transcription factor in the mid G1 phase. This leads to upregulation of Cyclin E transcription and other essential genes in the G1–S transition [132,133,134]. Furthermore, cyclin E can bind CDK2 and phosphorylate Rb in the late G1 phase to regulate its own expression through a positive feedback loop [135].

Molecularly, the G1 phase of the cell cycle is characterized by progressive increases in pRb, Cdt1 (protein involved in the formation of prereplication complexes), and Cyclin E. In conjunction, the levels of geminin (inhibitor of DNA replication), Cyclin A2 (protein that binds CDK1 and prevents cells from exiting the M phase), Cyclin B1 (marker of cell proliferation), and c-Myc (protein that activates cyclin and CDKs) decrease [136,137,138].

During the G1–S transition, Cyclins D1 and E predominate; however, during the S phase, Cyclin A levels increase and couple with CDK2 (predominant source of CDK in this phase). This is potentiated by the effects of CDC25A (cell division cycle 25 A), which is a protein phosphatase that activates CDK2 and is necessary for the G1–S transition [139].

During the S phase, the cell avoids re-replication of the DNA through degradation of Cdt1 (chromatin licensing and DNA replication factor 1) by E3 ubiquitin ligases, SCF^Skp2^ and CRL4^Cdt2^, or inhibition of Cdt1 by geminin. While Cyclin A/CDK2 complex levels continue to increase in the S phase, the level of Cyclin E decreases. Cells in the S phase can be detected by measuring BrdU (5-bromo-2-deoxyuridine) and Edu (5-Ethynyl-2′-deoxyuridine), which are thymidine analogs that incorporate into DNA in dividing cells [140,141].

Once the cell completes DNA replication, it enters into the G2 phase. In this phase, the expression of Cyclin A/CDK2 complexes is the highest. Cyclin B increases and complexes with CDK1 throughout the G2 phase. In addition, there is further reduction in Cyclin E and Cdt1. During this phase, PIK (phosphatidylinositol kinase) phosphorylates CDC25B and CDC25C phosphatases, which in turn activate the Cyclin A/CDK2 and Cyclin B/CDK1 complexes, respectively [142]. Once protein synthesis and cell growth are complete, the cell transitions into the M phase.

Prior to the G2–M transition, Cyclin B1/CDK1 complexes are inactive through Wee1 kinase-mediated phosphorylation [142]. During the G2–M transition, the levels of Cyclin B1/CDK1 complexes exceed a threshold; however, inhibition of CDK1 through Wee1-mediated phosphorylation prevents complex activation. During the transition, CDC25 removes inhibitory phosphates on CDK1. which allows cells to enter M phase [142]. During the M phase, Cyclin B/CDK1 complexes continue to rise and reaches peak levels. Other factors also increase, including PHH3 (phosphorylated histone H3), c-Myc, pRB, and geminin. During M phase, Cyclin E continues to decrease and Cyclin D1 increases [140,143].

Towards the end of M phase, the ubiquitin ligase complex APC/C (anaphase-promoting complex/cyclosome) and its coactivator CDC20 (cell division cycle 20) initiate the metaphase–anaphase transition by assembling ubiquitin chains that target Cyclin B1 and securin for destruction [144]. The degradation of securin activates separase, which cleaves a protein complex important for chromatid cohesion called cohesin. Subsequently, the cleavage of cohesin enables the separation of sister chromatids during anaphase and completion of mitosis [145]. APC/C also initiates degradation of Cyclin B1 and other important cell cycle regulators, which triggers mitotic exit and re-entry into and maintenance in the G1 phase [145]. In addition, the APC/C^CDC20^ complex may also target geminin for degradation, preventing DNA replication until the S phase [146,147]. When APC/C engages with adapter protein Cdh1 (cadherin 1), it may also limit the accumulation of mitotic cyclins in G1 that prevent premature entry into the S phase [144].

Throughout this process, cells can exit the cell cycle and enter into a resting state (G0) prior to re-entering the cell cycle at G1. Scientific knowledge of this quiescent state is scarce; however, some researchers have been able to characterize this phase through single-cell methods (e.g., time-lapse microscopy and immunofluorescence with automated image processing and cell tracking). During G0, there is an increase in the production of Cyclin E, p21, and Cyclin D1 and reductions in pRB and Cdt1. In G0, DNA content also returns to normal levels when compared to cells in the S or M phases, which is consistent with the reductions in BrdU and EdU seen in the G0 phase [140,148].

### 5.2. Cell Cycle Checkpoints after Radiation

**Figure 5:**
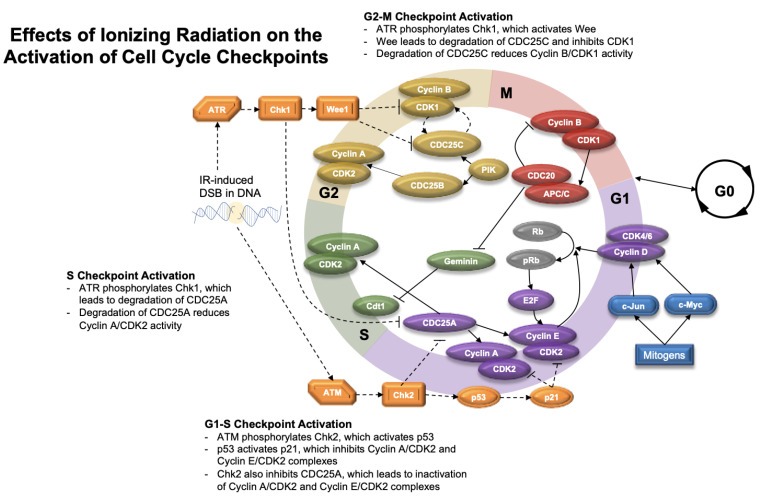
Diagram of radiation effects on the cell cycle. This diagram shows how ionizing radiation (IR)-induced double-stranded DNA breaks (DSB) initiate ATR (Ataxia-telangiectasia and Rad3 related protein) and ATM (ataxia-telangiectasia mutated) mediated activation of Chk1 (checkpoint protein 1) and Chk2 (checkpoint protein 2) protein kinases, respectively. Activation of Chk1 and Chk2 protein kinases leads to downstream events that push cells into cell cycle arrest to allow for DNA repair to occur. Dotted lines indicate events associated with radiation-induced changes to the cell cycle. APC/C (anaphase promoting complex), CDC25 (cell division cycle 25), CDK1/2/4/6 (cyclin-dependent kinase 1/2/4/6), Cdt1 (chromatin licensing and DNA replicating factor 1), E2F (family of transcription factors), p21 (cyclin dependent kinase inhibitor 1), p53 (tumor protein p53), PIK (phosphatidylinositol kinase), pRB (phosphorylated retinoblastoma protein), Rb (retinoblastoma protein), Wee1 (Wee1-like checkpoint kinase).

Cell cycle checkpoints exist throughout the cell cycle to monitor important events, such as cell size, DNA integrity, and segregation during mitosis. When radiation causes significant DNA damage, cells can enter cell cycle arrest at these specific checkpoints in order to repair injury (Figure 5).

The G1 checkpoint (i.e., the restriction point) commits the cell to cycle progression [54]. In response to DNA DSBs, ATM kinase is activated and phosphorylates Chk2 [149]. Chk2 inhibits CDC25A and serves as a crucial step in the G1–S checkpoint; CDC25A normally functions to disinhibit Cyclin A/CDK2 and Cyclin E/CDK2 complexes via dephosphorylation [150]. ATM is also responsible for the induction and stabilization of p53, which activates p21. Subsequently, p21 inhibits Cyclin A/CDK2 and Cyclin E/CDK2 complexes and promotes cell cycle arrest, further activating the G1–S checkpoint [151].

At the S checkpoint, ATR Kinase responds to DNA damage by activating Chk1. Chk1 activity leads to the degradation of CDC25A, thereby reducing Cyclin A/CDK2 complex activity, and halting DNA replication [151]. The G2–M checkpoint blocks DNA-damaged cells from progressing to mitosis and is also regulated by ATR and Chk1. Chk1 stimulates Wee1 kinase, which can promote degradation of CDC25C and inhibition of CDK1, which is important for progression to mitosis [54,151,152]. In addition, Chk1 activity blocks the positive feedback loop that occurs between CDK1 and CDC25C [142].

## 6. General Mechanisms of Radiation Resistance

Radiation therapy can initiate DNA damage. When DNA is not adequately repaired, cell cycle checkpoints can be activated and cells may enter cell cycle arrests in the G1, S, and G2/M phases, as described above [153,154]. Furthermore, in the S phase, relatively high doses of IR can directly injure replication machinery that also halts DNA replication [155]. When radiation-induced DNA damage is not adequately repaired while the cell is arrested, normal cells may undergo cell death.

Depending on the cell type and the phase of the cell cycle at time of irradiation, cells can express varying degrees of radiation resistance. Irradiated cells in the G1 phase are generally understood as being more radiosensitive; however, in some cell types, irradiated cells in the G1 phase may be more radioresistant because they can prolong the G1 phase to allow for DNA repair prior to transition to the S phase [156,157]. Irradiated cells in the late S phase may be more prone to radiation resistance because there is a second copy of DNA available for HR, if necessary, which can lead to the activation of robust DNA repair mechanisms [158]. Cells that are exposed to radiation in the G2/M phase of the cell cycle are most susceptible to radiation injury because of limited time for DNA repair prior to separation of sister chromatids [156]. However, cells may develop mechanisms to resist radiation injury by arresting in the G2/M phase for prolonged periods of time to allow for DNA repair [159].

Radiation-resistant cells may acquire adaptive features that allow them to: (1) repair DNA, (2) bypass normal cell cycle checkpoints, and (3) continue to replicate despite DNA damage. Repair of DNA damage relies on both DNA damage sensors and the DDR proteins, which are important to maintain genomic integrity and avoid activation of cell death mechanisms. Several radiation-resistant malignancies have evolved more efficient DNA repair mechanisms through the upregulation of DNA damage sensors and repair proteins.

Several radiation-resistant tumors have demonstrated overexpression of DNA sensor proteins, including BRCA1, Ku70/80, and Nbs1/Mre11/Rad50 complex and its components [76,160,161,162,163,164]. Other tumors can evade radiation injury by upregulating DNA repair proteins, such as RAD51, DNA-PK [160,165], RPA1 [166], LIG4 [167], HIF-1 [168], HDAC [169], Wee1 [170], CDK1 [171], and Chk1 [160,165,172,173,174]. In an in vitro study using normal human Schwann cells and merlin-deficient Schwann cells (MD-SCs), MD-SCs produced a robust RAD51 response when exposed to 6 Gy of radiation when compared to normal Schwann cells [175]. These findings suggest a possible mechanism of radiation resistance in schwannomas that warrants further investigation.

There are also limitations of the cell cycle checkpoints that allow cells to transition to the next phase despite radiation-induced DSBs. Irradiated cells in the G1 phase may be able to progress into the S phase for 4–6 h before the G1–S checkpoint is fully activated, albeit at a slower rate. The cells that transition into the G2 phase before the G1–S checkpoint is complete can progress through the cell cycle but demonstrate higher levels of DSBs [176]. In addition, cells with damage to or deficiencies in the players involved in the S-phase checkpoint can continue to replicate DNA in the presence of DSBs, a process termed radioresistant DNA synthesis (RDS). RDS can continue as the phase progresses, peaking in the latter part of the S phase [177]. After IR, cells may exit G2 arrest if the number of DSBs drops below a defined threshold (thought to range from 10 to 20 DSBs), allowing cells to enter the M phase before DNA repair is complete; however, it is unclear whether these cells are likely to continue proliferating [176]. Thus, radiation-resistant cells may progress through the cell cycle and continue to replicate due to natural limitations in the cell cycle checkpoints. In addition, cells may also express alterations in cell cycle proteins such as lower levels of CDK inhibitors, e.g., p21 and p27, which makes them more resistant to radiation [177].

When radiation-resistant cells bypass normal cell cycle checkpoints, they accumulate DSBs and chromosomal instability that may initiate cell death pathways. Radioresistant cells can have abnormal expression of various oncogenes and tumor suppressor genes as well as developing alterations in cell death pathways that make them resistant to radiation and tumor formation [178].

One factor known to increase tumor cell resistance to radiation is the presence of activated oncogenes. Hence, there has been considerable interest in determining which genes mediate altered radiosensitivity in tumor cells. The ability of the *ras* oncogene to lead to radioresistance has been indicated through several independent lines of experimentation [179]. There are many described mechanisms of radioresistance in relation to altered expression of oncogenes or tumor suppressor genes.

Examples of activated oncogenes associated with radiation resistance include ras, raf, c-Myc, YAP (Yes-associated protein 1), and HER1/2 (human epidermal growth factor receptor 1 or 2) [172,179,180,181,182,183]. The inactivation of tumor suppressor genes can also promote resistance to radiation. Alterations of the tumor suppressor gene p53 have been associated with the radioresistance seen in numerous solid and hematopoietic cancers, as these cells can bypass the G1–S checkpoint and avoid cell death [184,185,186,187,188]. Radiation resistance in tumors such as prostate cancer has also demonstrated reduced expression of tumor suppressor PTEN (phosphatase and tensin homolog) [189].

Aberrations in normal cell death mechanisms can also allow tumors to resist radiation injury. Upregulation of survival proteins that counteract cell death, such as survivin, have been associated with radiation resistance [190]. Tumors that have constitutively activated NF-κB (nuclear factor kappa B) can also resist radiation damage by overexpression of downstream gene products that block apoptosis [191,192]. Alternatively, underexpression of apoptosis-related proteins, such as caspase-1, caspase-3, and AIF can also prevent normal cell death processes from occurring, thereby promoting radiation resistance in various tumors [193,194,195].

## 7. Radiobiology and Radiation Resistance in Vestibular Schwannoma

### 7.1. Radiation Response in Patients with Vestibular Schwannoma

In a 2019 analysis of the United States National Cancer Database (NCDB) by Leon and colleagues, approximately 27% of patients with VS were initially treated with SRS [196]. While many of these tumors are responsive to radiation treatment with limited side effects, a proportion of VS display varying degrees of radioresistance. These differences in responsiveness are seen even among tumors of comparable size and histology [178].

Overall, the progression free survival (PFS) after GammaKnife SRS is approximately 84–94% [197,198,199]. However, some studies have shown that larger VS tumors and those from NF2 patients have an overall lower rate of tumor control than those published for sporadic tumors that are small and medium sized [34,35,200].

In a retrospective review of 46 NF2 patients treated with GammaKnife SRS for 73 vestibular schwannomas using a median marginal dose of 12.9 Gy (range 10–14 Gy), Sun et al. found that 41% of tumors demonstrated partial tumor regression, 43% had stable disease, and 16% showed tumor enlargement at last follow-up with magnetic resonance imaging (MRI) [34]. Although the tumor control rate was 84%, the range of follow-up was 8–195 months (median of 109 months).

In a large retrospective investigation of 871 patients that underwent GammaKnife SRS as initial treatment for VS, Johnson et al. found the overall PFS to be 94% at 10 years [197]. Although the PFS was excellent at 10 years, the variability in tumor size, radiation doses, and duration of follow-up may confound the results. The median tumor volume was 0.9 cc (range, 0.02–36 cm^3^), the median margin dose was 13 Gy (range, 12–26 Gy), and the median follow-up was 5.2 years (range, 1–25 years) [197]. On subsequent analysis, the authors found the PFS to be worse with larger tumor volume.

Smith et al. conducted a retrospective analysis of 177 patients with VS who received GammaKnife SRS with a prescription dose of 12 Gy to the 50% isodose line [198]. They found that the 2-year and 4-year progression-free survival rates were 97% (95% CI: 94.0%, 100.0%) and 88% (95% CI: 81.2%, 95.0%), respectively. Although the authors reduced treatment variability by evaluating patients that received one standardized radiation protocol, the radiographic duration of follow up was only 29.4 months (95% CI: 21.6, 37.1 months). In addition, the tumor volume was fairly broad, with a median tumor volume of 0.43 cm^3^ (range of 0.01–9.00 cm^3^) [198].

In a meta-analysis comparing 2579 patients that received surgery and 875 patients that received GammaKnife SRS, the tumor recurrence rate was better in the surgery group (1.55%) than the tumor progression rate in the radiation group (9%) [199]. The average peripheral dose of radiation was 17.27 Gy, suggesting that this investigation likely included older studies using radiation doses >11–13 Gy to the margin of the tumor. The mean follow-up time was also approximately 24 months for both groups, which may lead to underestimations of the tumor control rate at 5 and 10- years.

It is important to note that in these investigations, irradiated tumors may represent a mix of growing and nongrowing VS. The majority of retrospective studies assessing PFS do not take into consideration the natural history of VS, where approximately 65–71% of newly diagnosed VS do not demonstrate active growth in the first 2–5 years after diagnosis [1,11]. A longitudinal study from Denmark evaluating 729 patients that underwent observation for their VS showed that 17% of VS located in the internal auditory canal grew to involve the cerebellopontine angle and 28.9% of VS involving the cerebellopontine angle demonstrated growth of >2 mm in the largest diameter [1]. They also found that in tumors that grew after diagnosis, growth occurred in the first 5 years of the observation period, which serves as an argument that postirradiation VS studies should have radiographic follow-up of at least 5 years to prove radiation effectiveness. Pseudoprogression can also occurs in the first 18 months after radiation in approximately 23–44% of irradiated VS [23,24,25], which is another reason for longitudinal studies on VS to extend duration of follow-up beyond this period.

### 7.2. Tumor Growth Rate and Radiation Resistance in Vestibular Schwannoma

Although higher doses of single fraction radiation (e.g., 16–20 Gy to the 50% isodose line) are likely more effective at tumor control in VS, the higher rate of side effects has led to the adoption of more modern dosing strategies, with single fraction radiation (~11–13 Gy to the 50% isodose line) being the most commonly reported, followed by hypofractionated and fractionated protocols [15,18,201,202,203,204]. However, huge variabilities in patient selection and radiation protocols across studies prevent reasonable comparisons of single fraction radiation using GammaKnife SRS with hypofractionated or fractionated protocols using other linear accelerator (LINAC)-based systems, such as CyberKnife. In addition, variability in duration of follow-up and timing of radiographic follow-up, lack of uniformity in measuring outcomes (e.g., hearing loss, tumor growth) and statistical methods, and limited information on treatment adherence as it relates to side effect profile affect interpretation of clinical investigations published on radiation response of VS.

Beyond radiation dosing, little is known about the radiobiology of VS, why some tumors are more responsive to radiation than others, and how fractionation may affect tumor control. Based on the understanding of radiation biology in other cell types, it has been theorized that slower-growing tumors are less responsive to radiation than faster-growing tumors. This is from the understanding that proliferating cells are more sensitive to radiation than quiescent cells [156,203].

Although there is no standard definition for “fast growing” tumors, recent studies in patients with radiographic VS growth, on the contrary, showed that faster growing tumors are less responsive to radiation than slow growing tumors. Langenhuizen and colleagues performed an analysis of 311 patients with growing VS and stratified tumors into slow- and fast-growing categories based on tumor volume doubling time (<15 months versus >15 months, respectively). A total of 35 patients failed GammaKnife SRS. Kaplan–Meier analysis demonstrated that the estimated 10-year tumor control rates after SRS for fast- and slow-growing VS were 67.6% and 86.0%, respectively, suggesting that fast-growing tumors were less responsive than slow-growing tumors. Marston et al. conducted a retrospective investigation of 68 patients that received SRS after an initial observation period for a growing VS (>2 mm/year) and found that patients with pretreatment growth rates of <2.5 mm/year had a significantly higher tumor control rate (97%) than those with pretreatment growth of >2.5 mm/year (69%) [31]. Furthermore, in a retrospective study of 58 non-NF2 patients with VS, Niu et al. showed that slower volumetric tumor growth rates was a predictor of no postirradiation tumor expansion (i.e., >20% volumetric growth) [32]. In this study, VS tumors were treated with single fraction and fractionated radiation protocols. The authors showed that VS tumors with postirradiation tumor expansion had a median preirradiation growth rate of 89% per year, while VS tumors without postirradiation expansion had a median preirradiation growth rate of 41% per year [32]. Because of the heterogeneity in the radiation protocol, patient selection, tumor size and location, pretreatment growth rate, and duration of follow-up among published studies, comparing the growth rates of VS tumors that failed radiation to those that grew during the observation period would have inherent flaws.

**Figure 6:**
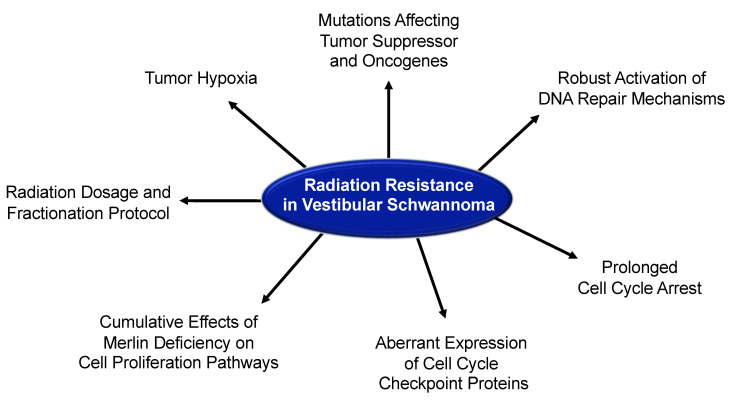
Potential mechanisms of radiation resistance in vestibular schwannomas. Radiation resistance may develop as a result of tumor hypoxia, mutations affecting tumor suppressor and oncogenes, robust activation of DNA repair mechanisms, prolonged cell cycle arrest, aberrant expression of cell cycle checkpoint proteins, cumulative effects of merlin deficiency on cell proliferation pathways, and/or radiation dosage and fractionation protocol used.

In the subsequent sections, the potential mechanisms underlying radiation resistance in VS are described and illustrated in Figure 6.

### 7.3. DNA Repair and Radiation Resistance in Vestibular Schwannoma

Although the molecular mechanisms for why faster-growing tumors would be less responsive to radiation are unknown, it was proposed that fast-growing tumors may be more radioresistant because they have efficient DNA repair mechanisms [30]. In an in vitro study, Cohen et al. found that MD-SCs had a more robust upregulation of DNA repair protein RAD51 after exposure to 6 Gy of radiation than normal Schwann cells, suggesting that VS may upregulate DNA repair mechanisms in order to resist radiation injury [175]. However, at higher doses of radiation (12 and 18 Gy single fraction), merlin-deficient Schwann cells did not activate RAD51 more than baseline, suggesting that higher doses of radiation may be required to prevent activation of DNA repair proteins. A recent study published by Thielhelm et al. found that radiation (18 Gy) induced the expression of gamma-H2AX, p21, and RAD51 in six cultured VS tumors, suggesting that irradiated VS acquire DSBs, can enter cell cycle arrest, and initiate RAD51 DNA repair in efforts to evade cell death [205]. In addition, three out of six cultured VS tumors were more resistant to 18 Gy of radiation and demonstrated more cell cycle arrest protein p21, when compared to 0 Gy and the three cultured VS that were more radiation responsive [205]. These findings suggest that radiation-resistant VS may mount a strong cell cycle checkpoint response, which may allow them to enter a prolonged state of cell cycle arrest to repair DNA DSBs. Further research into the activation of DNA repair mechanisms after irradiation in VS tumors may provide important insight on radiation resistance in VS and open avenues for testing radiosensitizers that target DNA repair.

### 7.4. Tumor Vasculature and Radiation Resistance in Vestibular Schwannoma

Because an important mechanism of radiation injury is the creation of ROS, it is possible that inadequate vasculature and tumor hypoxia may contribute to radioresistance in fast growing tumors [206,207]. VS tumors are known to express vascular endothelial growth factor (VEGF), a potent mediator of angiogenesis. In a retrospective investigation of 27 VS demonstrating tumor growth, Cayé-Thomasen et al. found that the concentration of VEGF expression and that of its high affinity receptor VEGFR1 on enzyme-linked immunoassay (ELISA) was correlated to tumor growth rate [208]. Although VEGF expression on immunohistochemistry of 18 growing VS found similar results [209], the effect of VEGF expression on radiation resistance in VS is unknown. Gao et al. found that anti-VEGF treatment reduced microvessel density in a sciatic mouse model implanted with human HEI193 schwannoma cells and murine NF2^-/-^ Schwann cells [210]. In addition, anti-VEGF (B20-4.1.1) treatment also reduced vessel tortuosity and vessel diameter in a cranial mouse model of schwannoma implanted with the same cell line [210]. Furthermore, treatment with anti-VEGF and 5 Gy of radiation significantly reduced the tumor growth when compared to control or either treatment alone. These findings suggest that anti-VEGF may normalize vasculature in NF2-associated schwannomas, which may improve radiation efficacy by increasing O_2_ perfusion, generating more ROS, and producing more radiation-induced DNA damage.

However, Lee et al. analyzed specimens from four VS patients who received primary SRS followed by salvage microsurgical resection; their results revealed a lack of necrosis or scar formation in all four tumors [38]. Similarly, Yeung and colleagues analyzed four VS samples from patients that failed SRS; these samples also displayed an absence of necrosis [178]. Extensive vascular hyalinization was found in both studies. Although further investigations are warranted, vascular hyalinization may lead to luminal stenosis, tumor hypoxia, impairment of the radiation-induced oxidative stress response, and absence of necrotic cell death [211].

### 7.5. Merlin Deficiency and Radiation Resistance in Vestibular Schwannoma

Faster-growing tumors may have altered expression of tumor suppressor and oncogenes that contribute to their fast growth and resistance to radiation. Merlin is a tumor suppressor protein that mediates cell proliferation through contact inhibition [212]. In VS, mutations in the *NF2* gene on chromosome 22q12 cause deficiency or dysfunction of merlin, which leads to loss of contact inhibition and unregulated cell proliferation and tumorigenesis [212,213,214]. Normally, merlin colocalizes with receptor tyrosine kinases (RTK), such as ErbB2/ErbB3, epidermal growth factor receptor, and platelet derived growth factor receptor, and block several downstream pathways important for cell proliferation. Merlin deficiency can promote tumorigenesis through dysregulation of the mitogen-activated protein kinase (Ras/Raf/MEK/ERK), phosphoinositide 3-kinases and protein kinase B (PI3K/Akt), proto-oncogene nonreceptor tyrosine kinase Src and focal adhesion kinase (FAK), Rac family small GTPase1 (Rac1) and p21-activated kinases (PAK), β-catenin, c-Jun N-terminal kinase (JNK), and mammalian target of rapamycin (mTOR) pathways. Deficiencies in merlin can also promote cell proliferation by releasing merlin inhibition of Yes-associated protein 1 (YAP1) in the Hippo pathway [212,213].

In a retrospective investigation comparing 8 irradiated and 49 nonirradiated VS, Gugel et al. found that progressive NF2-associated VS after irradiation demonstrated downregulation of phosphatase and tensin homolog (PTEN) and upregulation of mTOR signaling. These findings suggest that NF2-associated VS may resist radiation by downregulating the tumor suppressor PTEN while promoting PI3K/Akt signaling and overexpression of mTOR [215,216]. Because PTEN can initiate cell cycle arrest by inhibition of cyclin D [217], downregulation of PTEN may lead to radiation resistance in VS by promoting cell cycle progression and cell proliferation. Thus, mutations that modulate PTEN and mTOR signaling may enhance radiation resistance in VS.

Hansen et al. found that primary VS cells were relatively resistant to radiation [203]. In their study, radiation doses greater than 20 Gy were required to induced cell death through apoptosis. When ErbB2 was inhibited with PD158780 or the trastuzumab monoclonal antibody, the proliferation rate significantly reduced in nonirradiated and irradiated VS cells (30 Gy and 40 Gy). Because ErbB2 promotes cyclin D1 expression in the cell cycle, these findings suggest that ErbB2 inhibition likely promotes cell cycle arrest through downregulation of cyclin D1 [218,219]. Hansen et al. also discovered that ErbB2 inhibition with trastuzumab significantly reduced radiation-induced apoptosis, and activation of ErbB2 using exogenous neuregulin 1 (Nrg1) showed an opposite response with increased proliferation and more radiation-induced apoptosis [203]. From their findings, they theorized that radiation resistance in VS cells may reflect low proliferative potential. Their theory contrasts other clinical studies that have suggested that faster-growing VS are more radiation resistant [30,31,32]; however, this discrepancy may reflect the ex vivo study design and higher radiation dosages used in experiments with VS cells [203].

Because of merlin inactivation, VS cells demonstrate persistent JNK activation [220]. JNK can directly phosphorylate CDC25C during the G2 phase of the cell cycle, which leads to Cyclin B/Cdk1 activation, progression to mitosis, and unregulated cell proliferation [221]. Although the effect of JNK on radiation resistance in VS tumors is unknown, JNK inhibition may halt tumor growth by blocking cell cycle progression. In a study using JNK inhibitors SP600125 (20 μM) and I-JIP (20 μM), Yue et al. found that JNK inhibitors increased oxidative stress in primary VS cells exposed to 30 Gy of radiation, as demonstrated by higher levels of ROS [222]. However, at 20 μM, neither JNK inhibitors initiated more apoptosis in VS cells after 20 Gy exposures. With a very high dose of I-JIP (50 μM), irradiated VS cells expressed significantly more apoptosis. Further investigations are warranted to determine if JNK inhibition may increase VS sensitivity to radiation.

Deregulation of the pRb–CDK pathway, described previously, may also be involved in radioresistance of some VS. In a microarray analysis of eight VS performed by Lasak et al., seven of eight VS tumors underexpressed CDK2, when compared to normal vestibular nerve. In addition, two of those eight tumors had less Rb expression [223]. Merlin deficiency can lead to activation of Rac1/PAK [212], and a reduction in Rb in VS may further promote Rac1/PAK signaling [224]. Similar to JNK signaling, Rac1/PAK signaling facilitates cell proliferation by activation of CyclinB/Cdk1 complexes in the G2 phase and progression to mitosis [225]. Targeting Rb and/or Rac1/PAK may reduce radiation resistance in VS, but more confirmatory investigations are needed.

## 8. Conclusions

The normal cell cycle is a very complex series of events that ultimately allows cells to grow and divide. Overall, this process is well-controlled and undergoes multiple checkpoints to ensure high quality DNA replication and cell cycle progression. Radiation can initiate DSBs in the DNA that can activate cell cycle checkpoints; however, unrepaired DNA damage can lead to genetic instability that results in cell arrest and/or cell death. Some tumors may have developed mechanisms to counteract radiation-induced damage such as efficient DNA repair mechanisms and altered expression of tumor suppressor and oncogenes that allow them to bypass these checkpoints.

The response of VS cells to IR-induced damage remains poorly understood. By understanding the interplay between IR-induced DNA damage, DDR, cell death, and both cell cycle progression and arrest, we can better understand treatment resistance. When the radiobiology of VS and mechanisms of radiation resistance are fully elucidated, we can individualize radiation protocols and trial adjuvant therapies that can prevent and overcome radiation resistance in VS. Furthermore, research into the radiobiology of VS may lead to the identification of new molecular targets and the development of target-directed therapies for radioresistance.
